# Acetylcholinesterase Inhibitors (AChEI's) for the treatment of visual hallucinations in schizophrenia: A review of the literature

**DOI:** 10.1186/1471-244X-10-69

**Published:** 2010-09-07

**Authors:** Sachin S Patel, Azizah Attard, Pamela Jacobsen, Sukhi Shergill

**Affiliations:** 1 National Psychosis Unit, South London and Maudsley NHS Foundation Trust, Bethlem Royal Hospital, Monks Orchard Rd, Beckenham, BR33BX, UK; 2 Institute of Psychiatry, Kings College London, De Crespigny Park, London, SE5 8AF, UK

## Abstract

**Background:**

Visual hallucinations occur in various neurological diseases, but are most prominent in Lewy body dementia, Parkinson's disease and schizophrenia. The lifetime prevalence of visual hallucinations in patients with schizophrenia is much more common than conventionally thought and ranges from 24% to 72%. Cortical acetylcholine (ACh) depletion has been associated with visual hallucinations; the level of depletion being related directly to the severity of the symptoms. Current understanding of neurobiological visual processing and research in diseases with reduced cholinergic function, suggests that AChEI's may prove beneficial in treating visual hallucinations. This offers the potential for targeted drug therapy of clinically symptomatic visual hallucinations in patients with schizophrenia using acetylcholinesterase inhibition.

**Methods:**

A systematic review was carried out investigating the evidence for the effects of AChEI's in treating visual hallucinations in Schizophrenia.

**Results:**

No evidence was found relating to the specific role of AChEI's in treating visual hallucinations in this patient group.

**Discussion:**

Given the use of AChEI's in targeted, symptom specific treatment in other neuropsychiatric disorders, it is surprising to find no related literature in schizophrenia patients. The use of AChEI's in schizophrenia has investigated effects on cognition primarily with non cognitive effects measured more broadly.

**Conclusions:**

We would suggest that more focused research into the effects of AChEI's on positive symptoms of schizophrenia, specifically visual hallucinations, is needed.

## Background

Visual hallucinations are a well recognised and distressing symptom in a variety of psychiatric disorders including psychotic illness and dementia. The pathophysiology of the visual hallucinatory experience remains unclear and available pharmacological treatments are disorder rather than symptom specific. Visual hallucinations occur in various neurological diseases, but are most prominent in Lewy body dementia, Parkinson's disease and schizophrenia. The lifetime prevalence of visual hallucinations in patients with schizophrenia is thought to be more common than conventionally thought and estimates range from 24% to 72% [1,2]. Both dopaminergic and cholinergic perturbation have been associated with visual hallucinations, although neither of these offers a parsimonious aetiological model. More recent interest has been associated with the role of cortical acetylcholine (ACh) depletion and it's association with visual hallucinations; the level of depletion being thought to relate directly to the severity of the symptoms.

As with many cognitive processes, it is not entirely clear how the brain processes elementary visual stimuli and converts them into meaningful percepts; often in conditions of high ambiguity. A mechanism for this process could offer a route into understanding the basis of visual hallucinatory experiences. An elegant solution to this problem is offered by neuro-computational models based on Bayesian statistical principles, conceptualising visual processing through a hierarchical system enabling the human brain to function in environments of uncertainty [3,4]. Ascending, stimulus driven (bottom-up) and descending, context driven (top-down) pathways combine in an iterative manner to produce an accurate visual experience of our surroundings. Thus higher centres form predictions in anticipation of ascending signals allowing the visual system to process large amounts of objectively ambiguous information into accurate percepts. Yu and Dayan's 2002 review of acetylcholine in cortical inference hypothesises that acetylcholine plays a pivotal neuro-modulatory role in this top-down/bottom-up model [5]. It is proposed that acetylcholine in the human brain modulates the interaction between top-down and bottom-up processing in determining appropriate neural representations for inputs with the levels of ACh reflecting the uncertainty associated with top-down information processing [5]. According to this theory, low levels of cortical ACh would result in the increased salience of top-down information whereas high levels would result in over-processing of bottom-up, stimulus driven information. Using this model, visual hallucinations in ACh depleted cortices could be experienced due to over processing of top down signal and the resulting undue confidence in experiencing percept in the absence of sufficient visual stimuli strength [5]. In addition, dopaminergic neuro-modulation of cholinergic function is also considered in this review and contemporary dopaminergic theories of schizophrenia may be integrated into this pathway to conceptualise the formation of visual hallucinations in schizophrenia [6].

Conditions with underlying cholinergic deficits and high rates of visual hallucinations include Alzheimer's disease, dementia with Lewy bodies and Parkinson's disease. Targeted drug therapy of the cholinergic system in these conditions in order to treat visual hallucinations has shown benefit with AChEI's [7-12]. Much of this evidence is from case series' and randomised control trials assessing the efficacy of AChEI's on non cognitive symptoms broadly, rather than visual hallucinations specifically.

Applying current understanding of the cholinergic system in visual pathways and the evidence that AChEI's are of use in treating visual hallucinations in the conditions mentioned, it would appear feasible that they could also be used in schizophrenia related visual hallucinations. In this article, we review the use of AChEI's for the targeted treatment of visual hallucinations in schizophrenia.

## Methods

MEDLINE and EMBASE searches were performed using the search terms, schizophrenia or schizoaffective disorder and visual hallucinations and acetylcholinesterase inhibitors or donepezil or Aricept^® ^or galantamine or Reminyl^® ^or rivastigmine or Exelon^®^. No time limits were set when searching.

As this preliminary search found few articles of interest, a second wider search was conducted omitting the terms schizophrenia or schizoaffective disorder. The resulting articles were then assessed on an individual basis independently by two authors. Only those articles which investigated, commented on or case reported the targeted treatment of visual hallucinations in schizophrenia with AChEI's were included.

## Results

Our preliminary search found 3 articles. All 3 were not relevant to our objectives and related to Charles Bonnet Syndrome, Parkinson's disease and hypothetical modelling of visual hallucinations.

Our second search yielded 205 articles. After removal of duplicates, we again found no articles of relevance to our objectives. The majority of articles related to visual hallucinations in dementing illnesses and Parkinson's disease; including case reports, reviews and RCT's. Additional file 1 gives an overview of our searches according to PRISMA (Preferred Reporting Items for Systematic Reviews and Meta-Analyses) guidelines.

## Discussion

The results show that there is no direct evidence in the literature assessing the use of AChEI's in treating visual hallucinations in Schizophrenia. Our review was deliberately narrow in keeping with our objectives in order to identify a symptom specific treatment.

Studies of AChEI's use in schizophrenia tend to focus on cognitive symptoms as the primary area of investigation and non cognitive symptoms as a secondary area; the later includes psychopathology such as hallucinations and delusions. A recent systematic review and meta-analysis of the use of AChEI's as an adjunctive therapy in schizophrenia and schizoaffective disorder reported on their effects on psychopathology [13]. The authors found no statistically significant effect on the total Positive and Negative Syndrome Scale (PANSS) scores in those treated with AChEI's. This conclusion however may reflect on the chronicity and general clinical stability of the patient's included in these studies with a resultant lack of psychopathology at baseline. There were also a relatively small number of suitable studies for inclusion in the meta-analysis and therefore positive effects may have been more difficult to detect.

Given the use of AChEI's in targeted, symptom specific treatment in other neuropsychiatric disorders, it is surprising to find no related literature in schizophrenia. Studies have understandably focused on the cognitive enhancing properties of this class of drug. We would suggest however that more focused research into the effects of AChEI's on positive symptoms of schizophrenia, specifically visual hallucinations, is needed.

## Conclusions

Complex treatment-resistant patients are often left with residual symptoms, even after compliance with adequate doses of antipsychotic medication. In the absence of the advent of a superior novel antipsychotic to clozapine, a symptom based approach based on the best available research in other clinical disorders may provide some guidance in adjunct therapy. Novel therapies falling outside usual clinical practice may be necessary, with careful monitoring, both of clinical efficacy and of toxicity. This review aims to highlight the need for careful consideration of using AChEI's in treating non cognitive symptoms of schizophrenia, more specifically, visual hallucinations. It certainly highlights the need for studies investigating the use of adjunctive AChEI's for targeting specific residual positive symptoms in addition to continued investigation of their effects on cognitive symptoms.

## Competing interests

Authors have no competing interests to declare that are relevant to the content of this submission.

## Authors' contributions

SS contributed to planning, supervision, writing, and analysis of the study. SP, PJ and AA each contributed to data collection, writing the manuscript and review of the literature.

All authors have read and approved the final manuscript.

## Pre-publication history

The pre-publication history for this paper can be accessed here:

http://www.biomedcentral.com/1471-244X/10/69/prepub

## Supplementary Material

Additional file 1 **Prisma Flow Diagram for review of literature**. Click here for file

## References

[B1] Cummings J Miller B Visual hallucinations: Clinical occurrence and use in differential diagnosis West J Med 1987 146 46 51 3825109 PMC1307180

[B2] Bracha HS Wolkowitz OM Karson CN Bigelow LB High prevalence of visual hallucinations in research subjects with chronic schizophrenia Am J Psychiatry 1989 146 526 528 2929755 10.1176/ajp.146.4.526

[B3] Kersten D Mamassian P Yuille A Object perception as bayesian inference Annu Rev Psychology 2004 55 271 304 10.1146/annurev.psych.55.090902.142005 14744217

[B4] Friston K A theory of cortical responses Phil Trans R Soc B 2005 360 815 836 10.1098/rstb.2005.1622 15937014 PMC1569488

[B5] Yu D Dayan P Acetylcholine in cortical inference Neural Networks 2002 15 719 730 10.1016/S0893-6080(02)00058-8 12371522

[B6] Collerton D Perry E McKeith I Why people see things that are not there: A novel Perception and Attention Deficit model for recurrent complex visual hallucinations Behavioral and Brain Sciences 2005 28 6 737 757 16372931 10.1017/S0140525X05000130

[B7] Henriksen AL St Dennis C Setter SM Tran JT Dementia with lewy bodies: therapeutic opportunities and pitfalls The Consultant Pharmacist 2006 21 7 563 75 16934009 10.4140/tcp.n.2006.563

[B8] Edwards K Royall D Hershey L Lichter D Hake A Farlow M Pasquier F Johnson S Efficacy and safety of galantamine in patients with dementia with Lewy bodies: a 24 week open-label study Dementia and Geriatric Cognitive Disorders 2007 23 6 401 5 10.1159/000101512 17409748

[B9] Fabbrini G Barbanti P Aurilia C Pauletti C Lenzi GL Meco G Donepezil in the treatment of hallucinations and delusions in Parkinson's disease Neurological Sciences 2004 23 1 41 43 10.1007/s100720200022 12111620

[B10] Cummings JL Cholinesterase Inhibitors: A new Class of Psychotropic Compounds Am J Psychiatry 2000 157 1 4-15 10.1176/ajp.157.1.4 10618007

[B11] Cummings JL Askin-Edgar S Evidence for Psychotropic Effects of Acetylcholinesterase Inhibitors CNS Drugs 2000 13 6 385 395 10.2165/00023210-200013060-00001

[B12] Bullock R Cameron A Rivastigmine for the treatment of dementia and visual hallucinations associated with Parkinson's disease: a case series Current Medical Research and Opinion 2002 18 5 258 64 10.1185/030079902125000813 12240787

[B13] Ribeiz SRI Bassitt DP Arrais JA Avila R Steffens DC Bottino CMC Cholinesterase Inhibitors as Adjunctive Therapy in Patients with Schizophrenia and Schizoaffective Disorder: A Review and Meta-Analysis of the Literature CNS Drugs 2010 24 4 303 317 10.2165/11530260-000000000-00000 20297855

