# Supplementary material for: Acetylcholinesterase Inhibitors (AChEI's) for the treatment of visual hallucinations in schizophrenia: A review of the literature
Source: BMC Psychiatry. 2010 Sep 7;10:69. doi: 10.1186/1471-244X-10-69 (PMC2940862; doi:10.1186/1471-244X-10-69)
Supplement: Additional file 1 — Prisma Flow Diagram for review of literature. [file 1471-244X-10-69-S1.DOC]

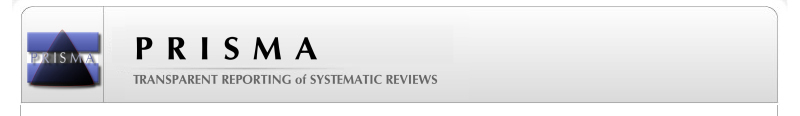
**PRISMA 2009 Flow Diagram**

**Screening**

**Included**

**Eligibility**

**Identification**

Records identified through database searching
(n =3 )

Additional records identified through other sources
(n = 0 )

Records after duplicates removed
(n =3 )

Records screened
(n =3 )

Records excluded
(n = 3 )

Full-text articles assessed for eligibility
(n =0 )

Full-text articles excluded, with reasons
(n = )

Studies included in qualitative synthesis
(n 0= )

Studies included in quantitative synthesis (meta-analysis)
(n =0 )
